# 

**Published:** 1890-06

**Authors:** 


### Bristol Medico-Chirurgical Journal.

### JUNE, 1890.

### CONTENTS.

ORIGINAL PAPERS: Page

Post-Partum Complications. By A. E. AUST LAWRENCE, M.D. ... ... 73

The Symptoms and Treatment of Outgrowths from the Back of the Tongue. By BARCLAY J. BARON, M.B. Edin.... ... ... ... ... ... 80

The Sequelæ of Suppurative Otitis Media. By F. H. EDGEWORTH, B.A., M.B. Cantab., B.Sc. Lond.... ... ... ... ... ... ... ... ... 87

On Some Points in the Surgical Physiology of the Abdomen and Pelvis. By T. S. ELLIS ... ... ... ... ... ... ... ... ... ... ... ... 98

CLINICAL RECORDS:

A Case of Acute Ascending Paralysis (Landry's Paralysis). By ARTHUR G. BLOMFIELD, M.D. Aberd. ... ... ... ... ... ... ... ... ... 113

Case of "Orbital Aneurism" Treated by Ligature of the Carotid. By A. W. PRICHARD, M.R.C.S. ... ... ... ... ... ... ... ... ... ... 115

Case of Epithelioma of the Body of the Uterus. By J. G. SWAYNE, M.D. 119

REVIEWS OF BOOKS ... ... ... ... ... ... ... ... ... ... ... ... ... ... 126

NOTES ON PREPARATIONS FOR THE SICK ... ... ... ... ... ... ... ... 134

PERISCOPE ... ... ... ... ... ... ... ... ... ... ... ... ... ... ... 137

SCRAPS ... ... ... ... ... ... ... ... ... ... ... ... ... ... ... 145

